# Hypoxia modifies the feeding preferences of *Drosophila*. Consequences for diet dependent hypoxic survival

**DOI:** 10.1186/1472-6793-10-8

**Published:** 2010-05-13

**Authors:** Paul Vigne, Christian Frelin

**Affiliations:** 1 CNRS UMR 6543, Univ Nice Sophia Antipolis, Nice, F-06108 France

## Abstract

**Background:**

Recent attention has been given to the relationships between diet, longevity, aging and resistance to various forms of stress. Flies do not simply ingest calories. They sense different concentrations of carbohydrate and protein macronutrients and they modify their feeding behavior in response to changes in dietary conditions. Chronic hypoxia is a major consequence of cardiovascular diseases. Dietary proteins have recently been shown to decrease the survival of chronically hypoxic *Drosophila*. Whether flies modify their feeding behavior in response to hypoxia is not currently known. This study uses the recently developed capillary feeding assay to analyze the feeding behavior of normoxic and chronically hypoxic *Drosophila melanogaster*.

**Results:**

The intakes rates of sucrose and yeast by normoxic or chronically hypoxic flies (5% O_2_) were analyzed under self selecting and "no choice" conditions. Chronically hypoxic flies fed on pure yeast diets or mixed diets under self selection conditions stopped feeding on yeast. Flies fed on mixed diets under "no choice" conditions reduced their food intakes. Hypoxia did not modify the adaptation of flies to diluted diets or to imbalanced diets. Mortality was assessed in parallel experiments. Dietary yeast had two distinct effects on hypoxic flies (i) a repellent action which eventually led to starvation and which was best observed in the absence of dietary sucrose and (ii) a toxic action which led to premature death. Finally we determined that hypoxic survivals were correlated to the intakes of sucrose, which suggested that dietary yeast killed flies by reducing their intake of sucrose. The feeding preferences of adult *Drosophila *were insensitive to NO scavengers, NO donor molecules and inhibitors of phosphodiesterases which are active on *Drosophila *larvae.

**Conclusion:**

Chronically hypoxic flies modify their feeding behavior. They avoid dietary yeast which appears to be toxic. Hypoxic survival is dependent on a source of exogenous sucrose. Ultimately, dietary yeast reduces hypoxic survival by reducing the intake of sucrose. The results highlight the importance of behavioral mechanisms in the responses of *Drosophila *to chronic hypoxic conditions.

## Background

Increasing interest has been devoted to the relationships between diet, longevity, aging and resistance to various forms of stress. For instance different protocols of dietary restriction increase longevity in model organisms such as *Drosophila melanogaster *and *Caenorhabditis elegans *and in mammals. There is still much controversy about the molecular and physiological mechanisms involved [1-3]. Recent studies have highlighted the importance of feeding behavior in dietary effects [4,5]. Flies do not simply ingest calories. They sense different concentrations of carbohydrate and protein macronutrients and they modify their feeding behavior in response to changes in dietary conditions.

Flies are usually fed with mixtures of carbohydrates and yeast which are provided as a semi solid, agar-based, mixture. There are two ways of presenting macronutrients to adult flies.

(i) "Self selection conditions ". Flies are exposed to separate sources of sucrose and yeast and are given the choice to select which food to ingest. Under these conditions, flies have a marked preference for sucrose over yeast [4,6].

(ii) "No choice conditions". Flies cannot select which nutrient to use and they are forced to ingest carbohydrates and proteins in defined proportions. Under these conditions, diluting the food mixture usually increases the longevity and the resistance of the flies to various forms of stress [2]. This effect is not due to calorie restriction *per se *[7,8]. It is mimicked by a selective yeast restriction [9-11] and it involves insulin dependent- [12] and independent pathways [1,13]. The proportions of carbohydrates and proteins ingested under "no choice" conditions may be widely different from the preferences of self selecting flies. As a consequence, flies have to compromise between eating one nutrient in excess to the other and under-eating others, relative to the intake target [14,15]. Flies usually maintain their intake of carbohydrates constant and abandon regulation of yeast intake [4,6]. These studies have highlighted the complexity of feeding behavior in simple organisms such as flies. More importantly, they have stressed the importance of assessing food intakes in all studies dealing with dietary effects.

*Drosophila *sense and adapt to low oxygen levels. Physiological adaptation to hypoxic conditions involves a variety of mechanisms that act at cellular, organ or organism levels [16-18]. We previously described that dietary proteins accelerate ageing of male *Drosophila *and decrease their survival under chronic hypoxic conditions [11,19]. Whether flies modify their feeding behavior in response to hypoxia is not currently known. Here, we used the recently developed capillary feeding assay [20,21] to analyze the feeding behavior of chronically hypoxic flies.

## Results

Laboratory strains of *Drosophila *are usually reared on mixtures of carbohydrates and yeast, which is used as a source of proteins. The feeding behaviors of chronically hypoxic (5% O_2_) and normoxic (21% O_2_) male flies were compared using selected diets and a capillary feeding assay. Food was provided by capillaries filled with pure sucrose (5S, 10S), pure yeast (5Y, 10Y) or mixed sucrose/yeast diets under "no choice" (5S5Y, 10S10Y) or self selection conditions (5S-5Y, 10S-10Y). The 8 feeding conditions allowed us to analyze the feeding preferences of the flies (sucrose *versus *yeast in self selection experiments), the influence of food presentation ("no choice" *versus *self selection conditions) and the capacity of the flies to get nutrients from diluted food sources (5% diets *versus *10% diets).

### Chronic hypoxia modified the feeding preferences

**Figure 1:**
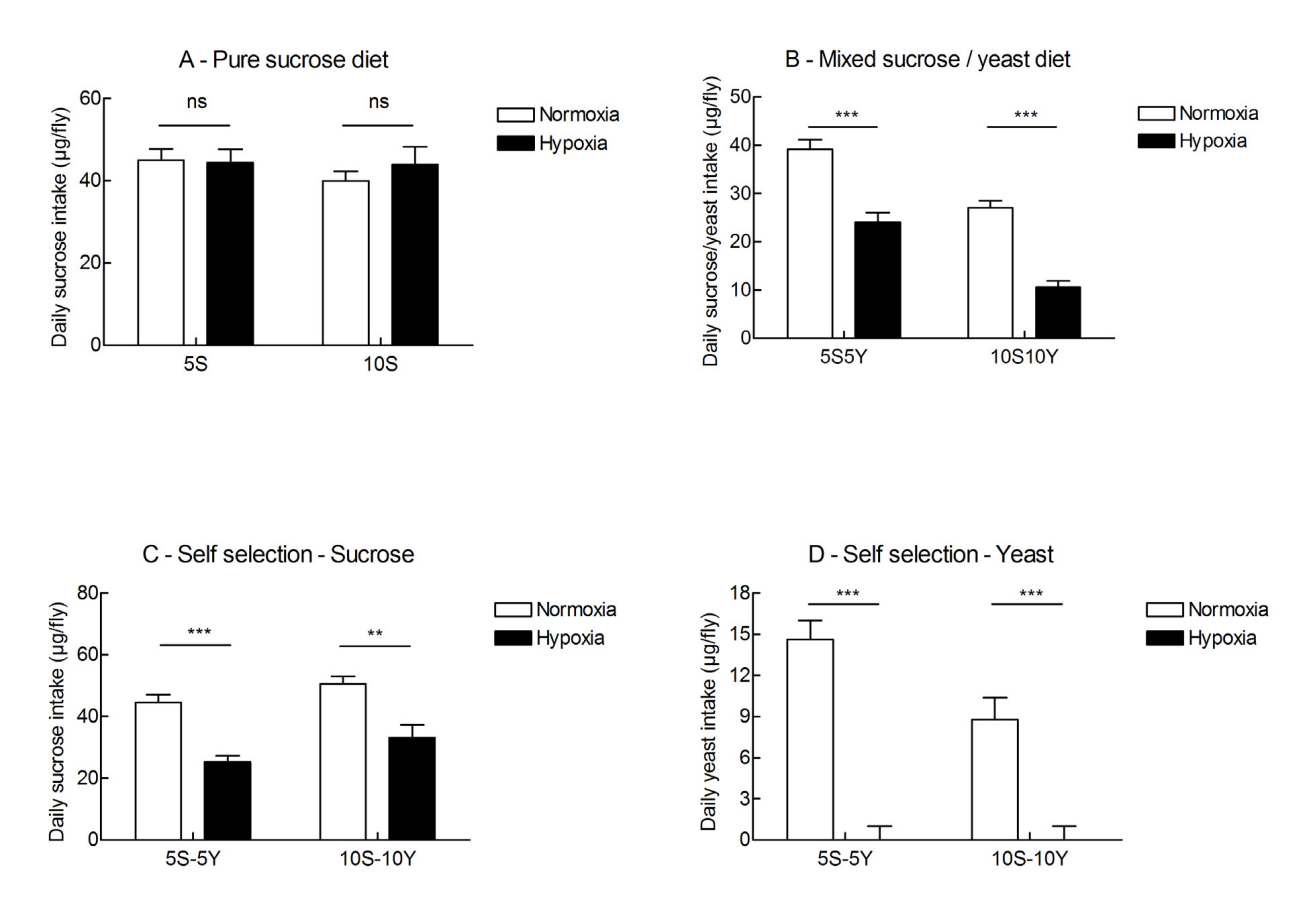
**Chronic hypoxia induced yeast avoidance**. Flies were fed on different diets as indicated under normoxic conditions (open bars) or chronically hypoxic conditions (filled bars). Daily nutrient intakes were measured using the capillary feeding assay. Mean daily intakes ± sem are indicated. Food intakes by normoxic and hypoxic flies were compared using unpaired t tests. ns: not statistically different, **: p < 0.05, ***: p < 0.01.

Figure 1 (panels A-C) compares the sucrose intakes by normoxic and chronically hypoxic flies fed under different conditions. Figure 1A shows that hypoxia did not change the intakes of sucrose when flies were fed on pure 5S or 10S diets. Under all other conditions, hypoxia decreased sucrose intake rates by 35-61% (Figure 1B and 1C, p < 0.01 in all comparisons).

Hypoxic flies fed on a source of yeast simply stopped feeding on yeast. This was observed both under conditions in which flies were fed on pure yeast solution (5Y or 10Y diets, data not shown) and under self selection conditions (5S-5Y and 10S-10Y diets, Figure 1D). Thus, hypoxic flies avoided feeding on yeast. It is important to note that hypoxic, self selecting flies which had the choice to feed on separate sources of sucrose and yeast stopped feeding on yeast and ingested sucrose (albeit at a slower rate than normoxic flies, Figure 1C and 1D). In contrasts, hypoxic flies fed on 1:1 mixtures of yeast and sucrose (5S5Y or 10S10Y diets), which could not select which food to use, did not stop feeding. Their net food intake was reduced by 39% (5S5Y diet) and 63% (10S10Y diet) as compared to normoxic flies fed on the same diets. These results indicated that hypoxia induced yeast avoidance and a decreased sucrose intake unless flies were fed on pure sucrose solutions.

### Chronic hypoxia did not change compensatory feeding

Compensatory feeding is one important aspect of the feeding behavior of *Drosophila*. Flies fed on diluted nutrient solutions usually increase their intake of solutions. This homeostatic mechanism allows flies to get an amount of nutrients that is independent of its concentration in nutrient solutions [6,20]. In this study we compared nutrient intakes by flies fed on diluted (5%) or concentrated (10%) sucrose solutions. Feeding compensation was defined as the ratio of intakes by flies fed on 10% and 5% sucrose solutions. A ratio of 2 indicated that flies did not compensate for the dilution of sucrose. A ratio of 1 indicated that flies compensated for food dilution. The compensation ratio was close to 1 when flies were fed on pure sucrose diets (normoxia: 0.9, hypoxia: 1,0). It was also close to 1 when flies were fed on separate sources of yeast and sucrose (normoxia: 1.1, hypoxia: 1.3). It was < 1 (normoxia: 0.7, hypoxia: 0.4) when flies were fed on mixed diet under "non choice" conditions. These results confirmed that feeding compensation was dependent on dietary conditions [6]. They further showed that hypoxia did not modify the capacity of the flies to adapt to dilute sucrose food sources.

### The response to imbalanced diets

**Figure 2:**
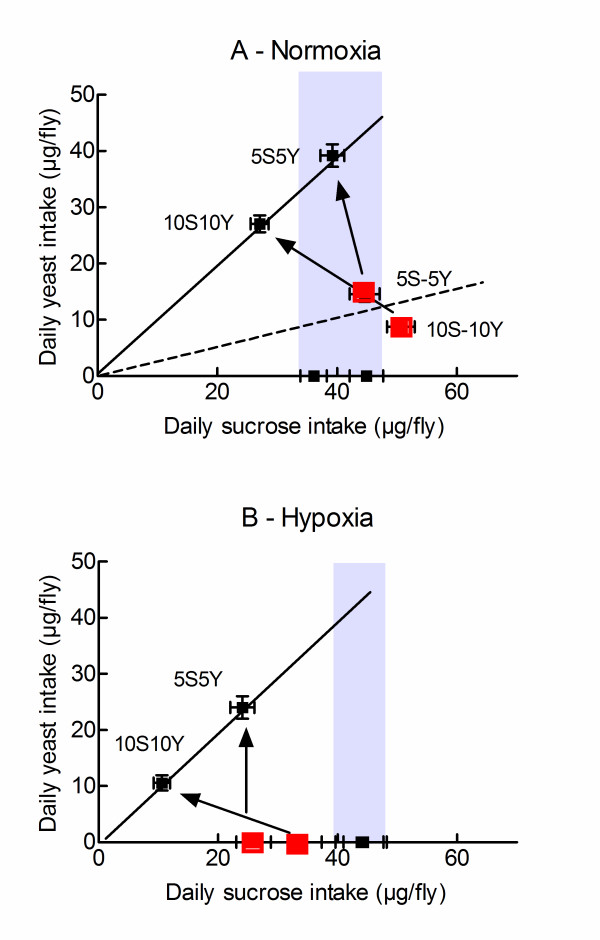
**Analysis of the feeding data using the nutritional geometrical framework**. Flies were exposed to different diets as indicated. The daily intakes of sucrose and yeast are plotted. A. Normoxic conditions. The dotted line shows the relationship that was expected if self selecting flies had a 4 fold greater preference for sucrose over yeast. The continuous line shows the relationship that was imposed by a mixed 1:1 yeast:sucrose diet under "no choice" conditions". The red squares indicate the feeding preferences of self selecting flies (5S-5Y and 10S-10Y diets as indicated). The grey zone indicates the range of sucrose intakes by flies fed on pure sucrose diets (5S or 10S diets). The arrows illustrate how flies adapt to an imbalanced 1:1 sucrose:yeast diet. All flies increased their intake of yeast as evidenced by an upward shift. Flies fed on a 10S10Y diet decreased their intake of sucrose as evidenced by a leftward shift. Flies fed on a 5S5Y diet did not. B, Hypoxic conditions. The same symbols as in panel A are used. The two panels are drawn at the same scale to favor comparisons. Note that flies fed on pure sucrose diets (5S, 10S) did not modify their intakes in response to hypoxia as evidenced by the relative positions of the grey areas in panels A and B. Hypoxia did not change the response of the flies to imbalanced 1:1 yeast:sucrose diets as documented by the similar orientations of the arrows. Hypoxia only modified the intake targets of self selecting flies (red symbols). Hypoxia induced yeast avoidance is not represented for clarity reasons.

The nutritional geometrical model is a useful representation to describe feeding preferences and the response of the flies to imbalanced diets [14,15]. Figure 2 uses this representation to compare nutrient intakes by normoxic and hypoxic flies. Figure 2A shows the yeast/sucrose diagram for normoxic flies. As previously described [4,6], self selecting, normoxic flies had a 4-fold preference for sucrose over yeast. Exposure of flies to mixed 5S5Y or 10S10Y diets forced them to ingest an imbalanced diet (1:1 yeast:sucrose) as compared to self selection conditions (1:4 yeast:sucrose). The responses of the flies are depicted as arrows in Figure 2A. Flies fed on 5S5Y diets increased their intake of yeast (p < 0.01) and did modify their intake of sucrose as compared to self selecting flies. Flies fed on 10S10Y diets increased their intake of yeast (p < 0.01) and decreased their intake of sucrose (p < 0.01) as compared to self selecting flies. Thus, normoxic flies prioritized their intake of sucrose and largely abandoned regulation of yeast intake.

Figure 2B shows that the yeast/sucrose diagram for hypoxic flies was different. As previously shown (Figure 1), self selecting flies stopped feeding on yeast and reduce their intake of sucrose. Hypoxia did not change sucrose intake by flies fed on pure sucrose diets. Finally, we observed that the responses of hypoxic flies to imbalanced diets were similar to the responses of normoxic flies. Hypoxic flies on a 5S5Y diet increased their intake of yeast (p < 0.01) and did not change their intake of sucrose as compared to self selecting flies. Hypoxic flies fed on a 10S10Y diet increased their intake of yeast (p < 0.01) and decreased their intake of sucrose (p < 0.01) as compared to self selecting flies.

Taken together these results indicated that hypoxia modified the feeding preferences of self selecting flies. It did not modify the responses of the flies to imbalanced diets.

### Dietary preferences and the survivorship of hypoxic flies

**Figure 3:**
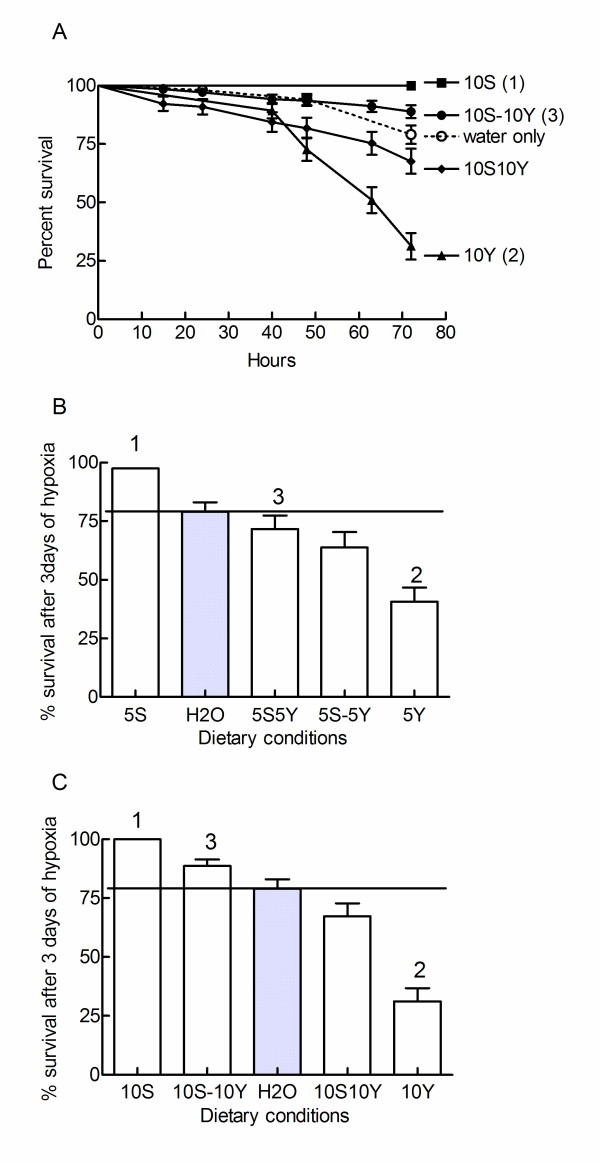
**Diet dependent hypoxic survival**. The survival of chronically hypoxic flies fed on different diets was monitored. Flies were fed with liquid diets provided by capillaries under the same conditions as feeding assays. A. Selected survivorship curves. Data were compared using the log rank test. (1) p < 0.0001 as compared to the water only condition, meaning that exogenous sucrose favored hypoxic survival. P < 0.0001 as compared to 10S10Y and 10S-10Y conditions, meaning that exposing flies to yeast decreased hypoxic survival. (2) p < 0.0001 as compared to water only, meaning that these flies did not die of starvation and that a yeast exposure was toxic to the flies. P = 0.0001 as compared to the 10S10Y condition, meaning that ingestion of sucrose limited yeast toxicity. (3) p = 0.0001 as compared to the 10S10Y condition, meaning that self selection conditions (and a reduced yeast intake) improved hypoxic survival. B, C. Survival after 72 hours of chronic hypoxia. The horizontal line and the grey bars indicate the survival of starving flies maintained on a source of water. Means ± sem are indicated. B. (1) p < 0.01 as compared to water only, p < 0.01 as compared to the 5S-5Y and 5S5Y conditions. (2) p < 0.01 as compared to all other conditions. (3) not different from the 5S-5Y condition. C. (1) p < 0.01 as compared to water only. (2) p < 0.01 as compared to all other conditions. (3) p < 0.01 as compared to the 10S10Y condition. Analysis of mortality data after 72 hours of hypoxia was consistent with the analysis of whole survival curves such as those presented in panel A. The number of flies used was 68-138.

The dramatic changes in feeding preferences documented by the previous experiments must have important consequences for the survival of the hypoxic flies. For instance, the starvation conditions induced by pure yeast diets is an obvious cause of hypoxic death. We therefore assessed the survivorship of hypoxic flies maintained under different feeding conditions. It is important to stress that survivorship and feeding assays were determined under the same experimental conditions. Data are thus fully comparable. Figure 3A shows selected survivorship curves. Figures 3B and 3C compare mortalities measured after 72 hours of hypoxia. Results can be summarized as follows.

1. Hypoxic flies fed on 5S or 10S diets lived longer than starving flies. This indicated that hypoxic survival was dependent on the exogenous supply of carbohydrates.

2. Addition of yeast to 5S or 10S diets under self selection or "no choice" conditions decreased longevity as compared to flies fed on pure sucrose diets. Thus, exposing flies to yeast decreased longevity. The influence of yeast was independent of the amount of yeast taken up by the flies. Flies on 5S-5Y or 10S-10S diets avoided feeding on yeast. Flies on 5S5Y or 10S10Y diets ingested large amounts of yeast (Figure 2).

3. Hypoxic flies fed on 5Y or 10Y diets avoided feeding and were shorter lived than hypoxic starving flies. This indicated that flies on pure yeast diets did not die of starvation and that the presence of yeast was toxic to them.

4. Food presentation modified hypoxic mortality. Feeding flies under self selecting conditions increased hypoxic survival as compared to "no choice" conditions, when flies were fed on rich, 10%, diets. This was not observed when flies were fed on poorer 5% diets.

5. Flies on mixed diets (5S5Y, 10S10Y, 5S-5Y and 10S-10Y conditions) were longer lived than flies exposed to pure yeast diets (5Y, 10Y). This indicated that ingestion of sucrose somehow limited the toxicity of yeast.

**Figure 4:**
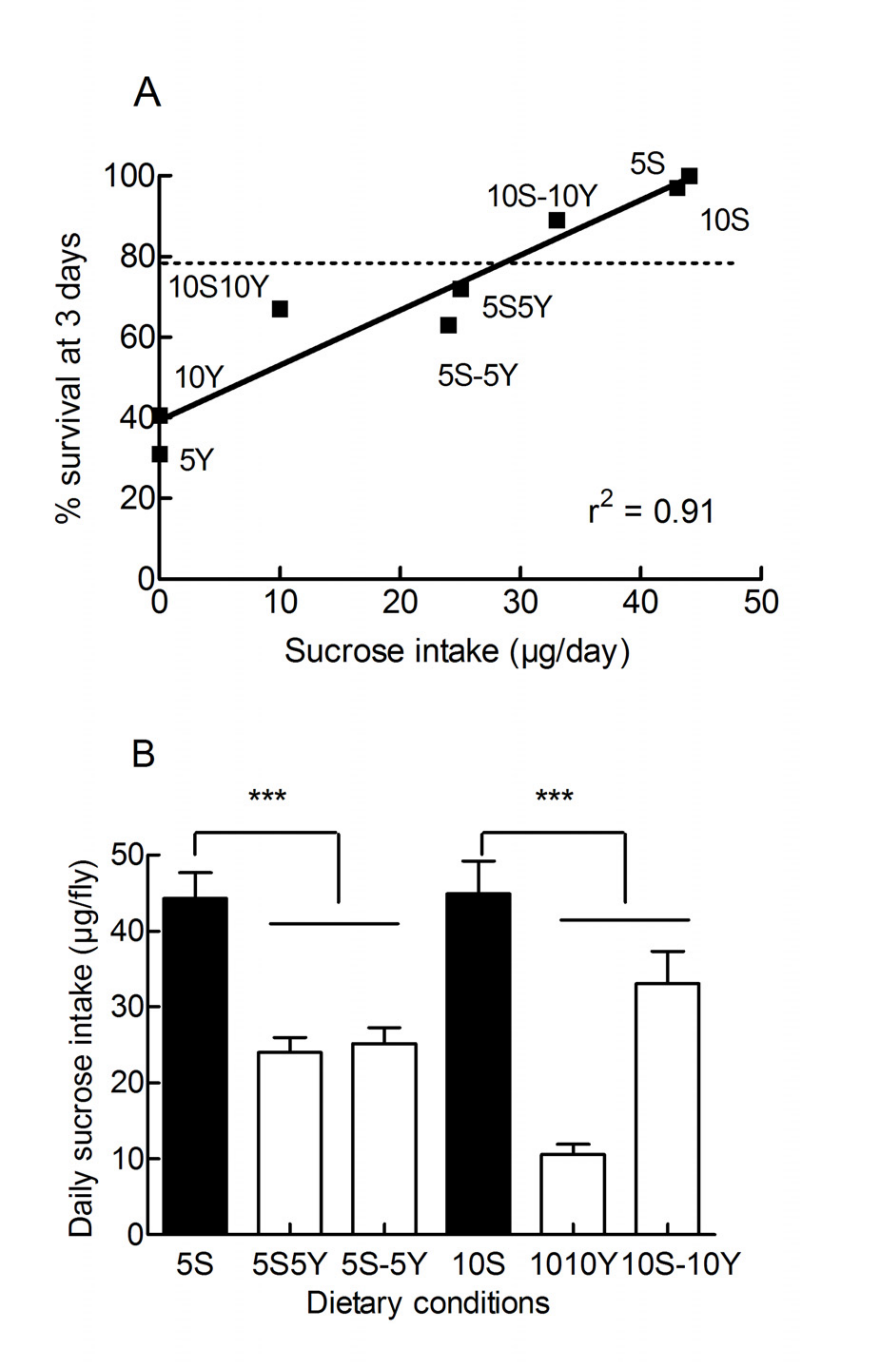
**Hypoxic survival of *Drosophila *correlates with sucrose intake**. A. Relationship between hypoxic survivals (measured after 72 hours of hypoxia) and daily sucrose ingestion. The dotted line shows the survival of starving flies exposed to a source of water. Only flies fed on 5S, 10S and 10S-10Y diets lived longer than starving flies. B. Exposure of flies to yeast reduced sucrose intake. Data presented in Figures 3B and 3C are reproduced to highlight the effect of yeast on sucrose intake. *** p < 0.01 as compared to pure sucrose conditions (filled bars)

Taken together these results indicated that yeast and sucrose interact in complex manners to determine the hypoxic longevity of the flies. We then tried to correlate hypoxic survival to the net intakes of yeast and sucrose. Surprisingly hypoxic tolerance was not related to yeast ingestion. For instance low yeast ingestion rates are associated to poor (5Y or 10Y diets), moderate (5S-5Y or 10S-10Y diets) or longer survivals (5S or 10S). In addition, flies which ingested the largest amounts of yeast (flies fed on 5S5Y or 10S10Y diets) were not the shortest living. In clear contrasts, hypoxic survival was strongly correlated to the daily intakes of sucrose (Figure 4A). Thus, exposing flies to a source of yeast reduced their survival (Figure 3B and 3C), but hypoxic survival was not correlated to yeast ingestion. One possibility for this result could be that yeast limited hypoxic survival by decreasing sucrose intake. Indeed, Figure 4B shows that yeast reduced sucrose intake by flies which did not ingest measurable amounts of yeast (e.g. flies on 5S-5Y or 10S-10Y diets) and by flies which ingested yeast (e.g. flies on 5S5Y or 10S10Y diets).

Taken together, these results indicated that hypoxic survival was dependent on the ingestion of sucrose and that an exposure to yeast extracts modified survival, probably by changing sucrose intake rates.

### The effect of pharmacological agents which modify NO signaling

A hypoxia dependent change in the behavior of *Drosophila *larvae has previously been reported. Larvae exposed to hypoxic conditions stop eating and begin exploratory behavior [22,23]. The mechanism involves a nitric oxide (NO) dependent formation of cyclic GMP. Exploratory behavior is induced by NO donor molecules such as Na nitroprusside under normoxic conditions. It is suppressed in hypoxic flies by NO scavenger molecules such as PTIO [22,23]. We therefore asked whether these pharmacological agents also modified the feeding behavior of adult flies.

In a first series of experiments, we tested the possible effects of sodium nitroprusside, a well known NO donor molecule. Normoxic flies were fed on a 5S5Y food mixture in the presence of 0.1 mM sodium nitroprusside. Total food consumption was identical in the two groups of flies (control: 0.84 ± 0.04 μl/day.fly, n = 4, treated: 0.79 ± 0.03 μl/day.fly, n = 7). A decreased consumption was expected. Sodium nitroprusside did not modify the feeding rates of normoxic flies fed on a 5Y diet (control: 0.98 ± 0.03 μl/day/fly, n = 4, treated: 0.87 ± 0.03 μl/day/fly, n = 7). Avoidance from yeast was expected. Sodium nitroprusside did not reduce the longevity of normoxic flies fed on a 5Y diet.

In a second series of experiments, normoxic flies were fed with a 5S5Y solution supplemented with 0.1 mM IBMX, an inhibitor of phosphodiesterases which degrade cyclic AMP and cyclic GMP into inactive AMP and GMP. Food consumption was not modified (control: 0.69 ± 0.10 μl/day/fly, n = 7, treated: 0.60 ± 0.04 μl/day/fly, n = 7). A decreased food intake was expected.

In a third series of experiment we fed hypoxic flies with a 5S5Y solution supplemented with 20 mM PTIO, a stable radical scavenger of endogenous NO. Ingestion of PTIO did not change food consumption (control: 0.38 ± 0.04 μl/day.fly, n = 6, treated: 0.34 ± 0.02 μl/day/fly, n = 6). An increase intake was expected if NO mediated the action of hypoxia.

In conclusion, available pharmacological evidence suggested that NO signaling did not contribute to hypoxia induced change in feeding behavior of adult flies.

## Discussion

The influence of chronic hypoxic conditions on feeding behavior was assessed at 5% O_2_. These conditions induce the expression of HIF-1/sima dependent reporter proteins in larval tissues [24]. They induce marked transcriptional responses in adults [25] and they decrease the survival of adult male flies in diet dependent and HIF-1/sima independent manner [26]. At lower oxygen tensions, adult flies reduce their metabolic rates; they switch to anaerobic metabolism and rapidly fall into stupor [27-29].

The feeding behavior of normoxic flies described in this study is fully consistent with previous reports [4,6,20]. It can be summarized as follows. (i) Flies have a marked, 4-fold preference for sucrose over yeast. (ii) Flies fed on diluted food solutions compensate for the dilution of their nutrients. They increase their intakes and get the same amount of nutrients as flies fed on more concentrated solutions. (iii) Flies fed on imbalanced 1:1 sucrose:yeast diets prioritize sucrose intake and ingest much more yeast than flies fed under self selection conditions.

Hypoxia changes the feeding preferences of the flies (see below). Hypoxia does not change the responses to diluted diets or to imbalanced diets. Thus hypoxia induces a specific change in feeding preferences. This study further defines the influence of macronutrients on hypoxic survival. Three major observations have been made.

(i) Yeast avoidance. Under hypoxic conditions, flies simply avoid feeding on yeast and this behavior can lead to a state of starvation. Yeast avoidance is observed when flies are exposed to pure yeast solutions in the absence or the presence of an additional source of sucrose. Flies do not stop feeding when yeast was mixed to sucrose. It is important to note that normoxic flies do not avoid yeast. Furthermore, dietary yeast is required for optimum survival [11]. Finally, yeast is well known to be an attractant for *Drosophila larvae*. Thus, yeast avoidance is a specific consequence of hypoxia.

(ii) Yeast toxicity. Flies fed on a pure yeast diet die sooner than starving flies. This suggests that starvation does not contribute to hypoxic mortality induced by yeast and that yeast is toxic by itself. It might appear surprising that flies fed on pure yeast diets do not ingest yeast and die of yeast exposure. One possibility could be that flies are sensitive to odorant substances from yeast extracts. For instance, Libert et al., [30] described that the longevity of flies under normoxic conditions is dependent on olfaction and food derived odors, including odors from yeast. It could also be that flies ingest minute amounts of yeast that cannot be measured using the capillary feeding assay and modify their behavior. We also observed that yeast toxicity is mainly observed when yeast is not mixed with sucrose. Yeast ingested together with sucrose in "no choice" experiments (5S5Y and 10S10Y diets) is not overtly toxic to the flies. This suggests that ingestion of sucrose probably relieves some of the toxicity of yeast. The mechanism of yeast toxicity has been analyzed in great details. It involves polyamines derived from dietary amino acids and the hypusination of initiation elongation factor 5A [26].

(iii) Sucrose and yeast macronutrients interact in complex and interrelated manners to determine hypoxic survival. Briefly, sucrose ingestion promotes hypoxic survival (Figure 4A) and limits the toxicity of yeast when sucrose/yeast mixtures are used. Exposing flies to yeast decreased sucrose intake (Figure 4B) and survival (Figure 3). The two actions of yeast do not require an ingestion of yeast. Taken together, these results suggest that, in the presence of sucrose, dietary yeast decrease hypoxic survival by limiting the intake of sucrose. In the absence of sucrose, yeast kills hypoxic flies.

Recent studies have drawn attention to the influence of the source of yeast on diet dependent feeding behavior and longevity. For instance, the beneficial action of dietary restriction on normoxic longevity is best observed when lyophilized yeast is used [5]. The yeast dependent hypoxic tolerance is observed in experiments which used both heat inactivated lyophilized yeast [19] and yeast extracts [26]. It is thus independent of the source of yeast. Ja *et al *[31] recently reported that water availability modifies the responses of flies to some protocols of dietary restriction. The flies used in our experiments are not water limited. They have access to a source of water that is independent of feeding capillaries. These are the best conditions to analyze the lifespan modulation by dietary restriction and the protein:carbohydrate ratio [30].

Preliminary pharmacological evidence presented here indicates that NO donor- and NO scavenger molecules which modify the exploratory behavior of hypoxic larvae [22,23], do not modify the feeding behavior of adult flies. This suggests that different mechanisms underlie the responses of larvae and adult flies to hypoxia. We cannot exclude the possibility, however, that larvae are more sensitive to drugs than adult flies. HIF-1/sima is a well known transcription factor that mediates many of the actions of hypoxia in vertebrates and invertebrates [16,17]. Oxygen dependent stabilization of an ODD-GFP reporter protein [16] and expression of HIF-1/sima dependent reporter proteins [24] have been reported in *Drosophila larvae*. The responses of adult flies are much weaker [24]. The possibility that HIF-1/sima dependent mechanisms contributed to hypoxia dependent feeding preferences is unlikely. No expression of HIF-1/sima dependent exogenous and endogenous reporter proteins could be observed under the conditions used in this study. A deeper hypoxia (1% O_2_) does however produce the expected responses [26].

Lastly, it is interesting to note that hypoxia dependent change in feeding behavior is not specific to *Drosophila melanogaster *and that similar mechanisms are probably operating in mammalian species. In man, exposure to high altitude (hypobaric hypoxia) is associated with a decreased food intake and a decrease in lean body weight [32,33]. Rats exposed to moderate hypobaric hypoxia reduce their food intake [34]. Finally, hypoxic rats under self selection conditions selectively decrease their intake of proteins [35]. Thus, the hypoxia dependent avoidance of dietary proteins is probably an evolutionarily conserved response to hypoxic conditions. Why should hypoxic flies avoid feeding on yeast? It is tempting to speculate that this behavioral mechanism protects organisms against the toxicity of dietary proteins which is uncovered by hypoxic conditions [26].

## Conclusion

The feeding behavior of flies is not fixed [4,20]. It is regulated by a variety of sensory stimuli such as olfaction and gustation, by metabolic needs, by nutrient signaling and by systemic internal signals of the feeding status such as insulin like peptides. The results presented here highlight the importance of behavioral mechanisms in the responses of *Drosophila *to chronic hypoxic conditions. Chronically hypoxic flies change their feeding behavior. They avoid dietary yeast which appears to be toxic. Flies on mixed diets reduce their food intake of nutrients and die. The *Drosophila *model might be useful to explore the genetic and neurological bases of these nutritional strategies. The capillary feeding assay can also be to screen pharmacological agents that modify feeding behavior and to further define regulations of feeding and macronutrient utilization under different environmental conditions.

## Methods

### Fly stocks

w^1118 ^flies (Bloomington Stock Center) were reared in 300 ml bottles filled with 30 ml of standard food medium (8.2% cornmeal, 6.2% sucrose, 1.7% heat inactivated baker's yeast and 1% agar supplemented with 3.75 g/l methyl 4-hydroxybenzoate) in humidified, temperature controlled chambers at 25°C and 60% relative humidity and under a 12:12 light: dark cycle.

### Capillary feeding assay

All experiments were performed using one day old male flies. Feeding behavior was assessed using a capillary feeding assay described previously [6]. Briefly, a piece of paper towel wetted with 2 ml of distilled water was introduced down to the bottom of 30 ml tubes. It had two functions. It provided a source of water that was independent of feeding and moist conditions to prevent evaporation of feeding solutions. Flies in groups of 6 were added to the tubes and tubes were sealed with rubber stoppers (SubA seal, Sigma/Aldrich, St Louis, Mo, USA) in which three holes had been drilled. Food was provided by one (or two) 75 μl micro capillary tube (Hirschmann Laborgeräte GmbH, Eberstadt, Germany). The third hole was used to insert a syringe needle for pressure compensation and gas exchanges. Capillaries were filled with 40 μl of liquid diet and inserted through the stopper via truncated 200 μl pipette tips. A mineral oil overlay (about 5 μl) was used to prevent evaporation from the top of the capillaries. In the "choice" experiments two capillaries were used. They were filled either with sucrose or yeast solutions and the flies were allowed to select which source of food to use. They will be called "self selecting flies". Only one capillary was used in the "no choice" experiments. It was filled with pure yeast, pure sucrose or mixed yeast/sucrose solutions. Flies were allowed to recover from CO_2 _anesthesia for 6 hours and maintained at 21°C. The height of the liquid column in the capillaries was measured twice a day using calipers and during 48 hours. Control experiments using fly free tubes were used to measure the rate of water evaporation under all conditions.

Nutrients were provided as water solutions of sucrose or of a yeast extract (Fluka ref. 70161, Sigma/Aldrich). Diets were labeled using the following conventions. A "10S10Y" diet means that flies were fed with a mixture of 10% sucrose and 10% yeast and had "no choice" to select their nutrients. A "10S-10Y" diet means that flies were exposed to separate sources of 10% sucrose and 10% yeast and could select which nutrient to consume. 10S and 10Y diets corresponded to 10% sucrose solutions and 10% yeast solutions respectively. 5S, 5Y and 5S5Y solutions were two fold dilutions of 10S, 10Y and 10S10Y solutions.

### Chemicals

Sodium nitroprusside, isobutylmethylxanthine (IBMX) and 2-phenyl-4,4,5,5-tetramethylimidazoline-1-oxyl 3-oxide (PTIO) and methyl 4-hydroxybenzoate were purchased from Sigma/Aldrich. Sodium nitroprusside and PTIO are water soluble and were dissolved into feeding solutions. IBMX (10 mM) was dissolved into ethanol and diluted 100 times in feeding solutions to obtain a final concentration of 0.1 mM. In these experiments, 1% ethanol was added to control feeding solutions. We confirmed that exposures of flies to 1% ethanol for less than 10 days did not modify their feeding behavior [21].

### Hypoxic conditions

Vials were inserted into the air lock of a "basic glove box" (PLAS LABS, Lansing, Mi, USA) maintained at a temperature of 21°C. The transfer chamber was flushed with a premixed 5% O_2 _atmosphere (Linde Gas). Vials were then transferred to the main chamber which had previously been equilibrated at 5% O_2_. The O_2 _and CO_2 _tensions in the main chamber were monitored using a Witt™ oxymeter (Witten, Germany). We checked that the O_2 _and CO_2 _tensions in the vials equilibrated with the atmosphere of the glove box. The large volume of the glove box ensured that O_2 _and CO_2 _tensions did not change during the time course of the experiments, for instance as a consequence of the metabolic activity of the flies.

Food consumption was measured without opening of the box, i.e. without reoxygenating flies. Dead flies were counted twice a day. Control normoxic experiments were performed at an ambient oxygen tension and were run in parallel to hypoxic experiments. Air pressure was atmospheric pressure (sea level).

### Data presentation and statistical analyses

Mean intakes of liquid (in microliters) were computed for each time point and using 10-13 independent experiments (and 6 flies in each experiment) and corrected for water evaporation. The relationship between net food intake and time was linear. The slope of the representation was calculated using least square regression. A student's t test statistics was then used to define whether the slope was different from zero and for comparing slopes obtained for different dietary conditions. P values < 0.01 were considered as statistically significant. Means ± sem are indicated. We checked that net nutrient intakes were linearly related to the number of flies used. Survivorship curves were compared using the log rank test. A z test was used to compare survivals of the flies after 72 hours of hypoxia. The GraphPad Prism 4 software was used for all statistical comparisons and for drawing the figures.

## Authors' contributions

PV and CF designed, performed and analyzed the experiments. CF wrote the manuscript. All authors read and approved the final manuscript.
